# Analysis of the relationship between perceived stress level and death anxiety in individuals with COPD

**DOI:** 10.1590/1980-220X-REEUSP-2023-0273en

**Published:** 2024-02-02

**Authors:** Zeliha Büyükbayram Genç

**Affiliations:** 1 Siirt University, Faculty of Health Sciences, Department of Nursing, Siirt, Turkey.

**Keywords:** Pulmonary Disease, Chronic Obstructive, Stress, Physiological, Anxiety, Enfermedad Pulmonar Obstructiva Crónica, Estrés Fisiológico, Ansiedad, Doença Pulmonar Obstrutiva Crônica, Estresse Fisiológico, Ansiedade

## Abstract

**Objectives:**

The study aimed to investigate the relationship between perceived stress level and death anxiety in individuals with COPD.

**Method:**

It was planned with a descriptive and relational screening design. It was carried out with the participation of 132 patients diagnosed with COPD. The study data were collected through Patient Information Form, Perceived Stress Scale, and Death Anxiety Scale. Descriptive statistics and multiple regression analysis were used in data analysis.

**Results:**

The COPD patients’ total perceived stress scale and perceived insufficient self-efficacy and perceived stress/distress subscale mean scores were found as 32.75 ± 5.32, 15.81 ± 3.60, and 16.93 ± 2.97, respectively. The patients’ Anxiety total scale mean score was determined to be 6.96 ± 3.40. A positive and statistically significant relationship was found between COPD patients’ Perceived Stress total scale mean score and their Death Anxiety Scale mean score (F = 4.332, p < 0.05).

**Conclusion:**

Perceived stress level of COPD patients was found to be at a high level, while their death anxiety level was determined as moderate. It was also determined that as perceived stress levels of the patients increased, their death anxiety levels also increased.

## RESUMEN

**Objetivos:**

Investigar la relación entre el nivel de estrés percibido y la ansiedad ante la muerte en personas con EPOC.

**Método:**

Se planificó con un diseño de screening descriptivo y relacional. En el estudio participaron 132 pacientes diagnosticados de EPOC. Los datos del estudio se recopilaron mediante el Formulario de información del paciente, la Escala de estrés percibido y la Escala de ansiedad ante la muerte. En el análisis de datos se utilizaron estadísticas descriptivas y análisis de regresión múltiple.

**Resultados:**

Las puntuaciones medias de la escala total de estrés percibido de los pacientes con EPOC y de la autoeficacia percibida insuficiente y de la subescala de estrés/angustia percibida fueron 32.75±5.32, 15.81±3.60 y 16.93±2.97 respectivamente. Se determinó que la puntuación media de la escala total de Ansiedad de los pacientes era 6.96 ± 3.40. Se encontró una relación positiva y estadísticamente significativa entre la puntuación media de la escala total de Estrés Percibido de los pacientes con EPOC y la puntuación media de la Escala de Ansiedad ante la Muerte (F = 4.332, p <0.05).

**Conclusión:**

Se descubrió que el nivel de estrés percibido por los pacientes con EPOC era alto, mientras que se determinó que su nivel de ansiedad ante la muerte era moderado. También se determinó que a medida que aumentaban los niveles de estrés percibido de los pacientes, también aumentaban sus niveles de ansiedad ante la muerte.

## RESUMO

**Objetivos:**

Objetivou-se investigar a relação entre nível de estresse percebido e ansiedade de morte em indivíduos com DPOC.

**Método:**

Foi planejado com design de triagem descritiva e relacional. Foi realizado com a participação de 132 pacientes diagnosticados com DPOC. Os dados do estudo foram coletados por meio da Ficha de Informações do Paciente, da Escala de Estresse Percebido e da Escala de Ansiedade de Morte. Estatísticas descritivas e análises de regressão múltipla foram utilizadas na análise dos dados.

**Resultados:**

Os escores médios da escala total de estresse percebido e autoeficácia percebida insuficiente e estresse/angústia percebida dos pacientes com DPOC foram de 32.75 ± 5.32, 15.81 ± 3.60 e 16.93 ± 2.97, respectivamente. A pontuação média da escala total de ansiedade dos pacientes foi determinada em 6.96±3.40. Foi encontrada uma relação positiva e estatisticamente significativa entre a pontuação média da escala total de Estresse Percebido dos pacientes com DPOC e a pontuação média da Escala de Ansiedade da Morte (F = 4.332, p < 0.05).

**Conclusão:**

O nível de estresse percebido dos pacientes com DPOC foi considerado alto, enquanto o nível de ansiedade de morte foi considerado moderado. Também foi determinado que, à medida que os níveis de estresse percebidos pelos pacientes aumentavam, os níveis de ansiedade de morte também aumentavam.

## INTRODUCTION

Chronic Obstructive Pulmonary Disease (COPD) is a chronic disease with no full recovery and characterized by the obstruction of airflow in the lungs^(1)^. According to the World Health Organization (WHO) data, COPD affects nearly 300 million individuals worldwide. COPD has been reported to be the third leading cause of death, with 3.23 million deaths in 2019 across the world^(2)^. It is the third leading cause of death related to respiratory system diseases in Turkey, accounting for 61.5% of these deaths^(3)^.

COPD mostly occurs in the middle and advanced age group and progresses slowly. The symptoms of the disease usually increase in winter months. This increase is a serious cause of morbidity and mortality^(4)^. Therefore, increased COPD cases occupy a significant place globally. In addition, many restrictions and physical-psychosocial problems are experienced by individuals. Due to these problems brought about by COPD, individuals with COPD are also mentally negatively affected. This situation lays the ground for the development of stress and death anxiety in individuals affected^(1,5,6)^.

Perceived stress is the individual’s perception regarding to what degree a situation or an event is threatening and out of his/her control^(7)^. In addition to difficulty in breathing in COPD, many situations, such as continuous health checks, oxygen use, and medication use, cause patients to experience stress^(4,6,8)^. High level of perceived stress in patients is seen as an obstacle to ensuring effective symptom management^(9,10)^. Stress also aggravates the individual’s compliance to treatment^(6,11)^. If the individual cannot adapt to the developing situation, s/he can experience many adversities such as death anxiety^(9,11)^.

It is important to reduce perceived stress levels in terms of coping with death anxiety, which is one of the psychological problems that can be experienced by individuals with COPD. COPD patients were asked about the feelings they experienced during dyspnea, and most of them expressed that living with COPD was highly difficult, and that they were scared of dying^(1)^. In a study conducted with another group, it was determined that patients experienced death anxiety^(11)^. Besides, determining perceived stress levels in individuals with COPD and ensuring their compliance with treatment should be part of the care provided by nurses. To provide comprehensive care to the individual, nurses should handle him/her with his/her physical, psychological, sociocultural, and economic aspects, be informed about the disease, and plan the care specifically for the individual and implement it^(1,12)^.

It is believed that the findings obtained in the study will be beneficial in terms of revealing perceived stress and death anxiety levels in individuals with COPD. In the literature review, stress^(8,9)^ and death anxiety^(9,11)^ were studied separately in a limited number of COPD patients, but since there was no study examining the perceived stress level and death anxiety level together, it is thought that the study will make a significant contribution to the literature in this regard. The primary aim of the study was to determine perceived stress and death anxiety levels in COPD patients, and the secondary aim was to investigate the relationship between perceived stress and death anxiety.

## METHOD

### Study Design

This is a descriptive, and relational screening design study, developed according to the guidelines of the Strengthening the Reporting of Observational Studies in Epidemiology (STROBE)^(13)^.

### Population of the Study

The study was conducted in the chest diseases inpatient clinics of Siirt training and research hospital between May 2022-April 2023. The hospital where the study was conducted is the only training and research hospital in the city and has 450 beds. It has clinics, outpatient clinics, and laboratories related with chest diseases (laboratory tests, respiratory function test, bronchoscopy, etc.). Various chest diseases can be diagnosed and treated in it. Numerous specialist doctors and nurses provide services related to chest diseases.

The population of the study was 420 patients diagnosed with COPD who received inpatient treatment at Siirt training and research hospital chest diseases clinic. It was conducted with 132 patients who met the research criteria within the determined process.

### Sample Criteria of the Study

The study sample size was calculated by using G*Power 3.1.9.7 software. As a result of the power analysis, the sample size was calculated as 120 patients, with 0.290 impact size, 95% power, and 0.05 margin of error^(14)^. The study was conducted with a total of 132 participants (considering an average of 10% participant loss) who met the inclusion criteria by convenience sampling method. A post hoc power analysis was performed to support the selected sample size^(14)^. The power of the study was calculated as 0.95%, with a moderate effect size (f^2^ = 0.295), at an ɑ value of 0.05, with the sample size of 132, five predictor variables, and squared multiple correlations r^2^ = 0.228 as inputs. These values show that the sample size was at the desired level.

### Inclusion and Exclusion Criteria

The patients who agreed to participate in the study were diagnosed with COPD for at least 6 months (diagnosed with ICD-10 International Classification of Disease-10 codes), were at the age of 18 years and above, and had no communication problems. Those who did not give their consent and had problems in communicating, seeing, or hearing were excluded from the study.

### Data Collection

Data were collected by the researchers through face-to-face interview technique. Each interview lasted approximately 10–15 minutes, and took place in a quiet environment, with questionnaires being administered when the patient felt well. It was estimated that the majority of the patients were illiterate or had a low literacy rate, and the researcher asked questions and recorded their answers.

### Data Collection Instruments

The study was performed through the Patient Information Form, Perceived Stress Scale, and Death Anxiety Scale Forms.

#### PERSONAL INFORMATION FORM

The form prepared by the researchers in line with the literature^(4,11)^ consists of 10 questions inquiring about the patients’ age, sex, marital status, education level, employment status, duration of COPD, other chronic diseases, having received training on the disease, smoking status, disease-related symptoms causing distress. In addition, it was asked whether the patient had sufficient knowledge about the disease by asking an educational question about COPD (basic information about COPD; medications, prevention, exacerbations, what to do in case of advanced disease, respiratory rehabilitation and exercise).

#### PERCEIVED STRESS SCALE (PSS)

The scale developed by Cohen et al.^(15)^ in 1983 consists of 14 items. The Turkish validity and reliability study of the scale was conducted by Eskin et al.^(16)^. The 5-point Likert type scale is scored between Never (0) and Very Often (4). Seven of the items which include positive statements (4, 5, 6, 7, 9, 10, and 13) are reversely scored. The scale consists of two subscales, which are Perceived Insufficient Self-Efficacy (items 4, 5, 6, 8, 9, 10, and 13) and Perceived Stress/Distress (items 1, 2, 3, 7, 11, 12, and 14). A total scale score between 0-13 is considered low level of perceived stress, between 14-27 a moderate level of perceived stress, between 28-41 a high level of perceived stress, and between 42-56 a very high level of perceived stress. High scores obtained from the scale indicate the individual’s high perceived stress level. The Cronbach’s alpha coefficient of the scale was determined to be 0.84^(16)^. In the present study, this coefficient was found as 0.76.

#### DEATH ANXIETY SCALE (DAS)

The scale was developed by Templer in 1970^(17)^. The Turkish validity and reliability study of the scale was conducted by Akca and Kose^(18)^. The 15-item scale is scored as Yes/No. Each “Yes” response given to the first 9 items on the scale gets “1” point, and “No” response gets “0” point, while each “No” response given to the other 6 items gets “1” point, and “Yes” response gets “0” point. Scores to be obtained from the scale between 0–4 show slight level of anxiety, between 5–9 moderate anxiety, between 10–14 high level of anxiety, and 15 indicates death anxiety at a panic level. The Cronbach’s alpha coefficient of the scale was found as 0.75^(18)^. In the present study, the Cronbach’s alpha coefficient of the scale was determined to be 0.74.

### Data Analysis

Data were analyzed in the statistical software SPSS 25 (Statistical Package for Social Science). Shapiro-Wilks Normality test was applied to determine whether the dependent variables showed a normal distribution according to their descriptive characteristics. In the analysis of the data, descriptive statistics (number, percentage, mean, and standard deviation) and multiple regression analysis were used. A statistically significant p < 0.05 value was accepted.

### Ethical Procedures

This study was carried out according to the Helsinki principles. Before starting the study, written permission from the hospital where the study was conducted and ethical approval from Siirt University Non-Invasive Clinical Research Ethics Committee (Date:26.04.2022, No: 42172) were obtained. In addition, written and verbal consent was obtained from the patients participating in the study.

## RESULTS

It was determined in the study that the patients’ mean age was 59.36 ± 11.51 years, 73.5% were male, 81.8% were married, 32.6% were illiterate, 68.2% were unemployed, 37.1% had a duration of the disease between 6–10 years, 59.8% had another chronic disease, 64.4% had received training on COPD, 61.4% quit smoking, 73.5% experienced dyspnea, 41.7% experienced cough, and 59.8% had phlegm (sputum) symptom (Table 1).

**Table 1:** Distribution of the patients by socio-demographic and disease characteristics (n:132) – Siirt, Southeast Region, Turkey, 2022.

Characteristics of the patients	Number (n)	%
**Sex**		
Female	35	26.5
Male	97	73.5
**Marital status**		
Married	108	81.8
Single	24	18.2
**Education level**		
Illiterate	43	32.6
Literate	33	25.0
Primary education	32	24.2
High school or higher	24	18.2
**Employment status**		
Yes	42	31.8
No	90	68.2
**Duration of COPD**		
0–5 years	47	35.6
6–10 years	49	37.1
11 years or more	36	27.3
**Other chronic diseases**		
Yes	79	59.8
No	53	40.2
**Getting education about the COPD disease (basic information about COPD)**		
Yes	85	64.4
No	47	35.6
**Smoking status**		
I’m drinking	24	18.2
Quit smoking	81	61.4
I never drank	27	20.5
**Disease-related symptoms**		
**Dyspnea**		
Yes	97	73.5
No	35	26.5
**Coughing**		
Yes	55	41.7
No	77	58.3
**Phlegm**		
Yes	79	59.8
No	53	40.2
**Others (respiratory tract infections, fatigue…etc)**		
Yes	89	67.4
No	43	32.6
**Age**	** $\bar{\text{X}}$ ± SD** **59.36 ± 11.51**	

SD: Standard deviation; $\bar{\text{X}}$ = Mean; Min: Minimum; Max: Maximum.

The total PSS score average of COPD patients was 32.75 ± 5.32, and perceived stress was found to be at a high level, while the total DAS score average was 6.96 ± 3.40, and death anxiety was found to be at a moderate level (Table 2).

**Table 2:** The patients’ mean scores obtained from perceived stress scale and death anxiety scale – Siirt, Southeast Region, Turkey, 2022.

Scale and subscales	Min.-Max. scores	$\bar{\text{X}}$ ± SD
**Total PSS and subscales**	17.00–49.00	32.75 ± 5.32
Insufficient/self-efficacy perception subscale	8.00–26.00	15.81 ± 3.60
Stress/distress perception subscale	9.00–27.00	16.93 ± 2.97
**Total DAS**	00.00–14.00	6.96 ± 3.40

SD: Standard deviation; $\bar{\text{X}}$ = Mean; Min: Minimum; Max: Maximum; PSS: Perceived stress scale; DAS: Death anxiety scale.

Multiple regression analysis was performed for the patients’ socio-demographic characteristics and PSS and its subscales, and DAS. In the analysis performed for PSS subscale of perceived insufficient self-efficacy, marital status, duration of the disease, and presence of another chronic disease were found to be statistically significant predictors (F = 7.028, p < 0.05). In the analysis performed for PSS subscale of perceived stress/distress, smoking status was determined to be a statistically significant predictor (F = 11.430, p < 0.05). In the analysis performed for PSS total scale, sex, presence of another chronic disease, and smoking status were found to be statistically significant predictors (F = 7.22, p < 0.05). Moreover, in the analysis performed for DAS total scale, marital status, employment status, having received training on the disease, and dyspnea symptom were determined to be statistically significant predictors (F = 7.456, p < 0.05) (Table 3).

**Table 3:** Regression analysis results in terms of descriptive characteristics (n:132) – Siirt, Southeast Region, Turkey, 2022.

Dependent variables		Independent variables	Unstandardized coefficients		Standardized coefficients	t	Sig.	95,0% CI	
			B	SE	*β*			Lower bound	Upper bound
**Insufficient/self-efficacy perception subscale**	1	(Constant)	13.230	.749		17.65	**000**	11.747	14.713
		Marital status (Married)	1.562	.771	.168	2.027	**.045**	.037	3.087
		Duration of COPD (11 years or more)	1.760	.673	.218	2.618	**.010**	.430	3.091
		Other chronic diseases (No)	2.067	.616	.282	3.353	**.001**	.847	3.286
		R = 0.376 R^2^ = .141 F = 7.028 p = .000*							
**Stress/distress perception subscale**	2	(Constant)	16.505	.279		59.09	**.000**	15.952	17.057
		Smoking status (I never drank)	2.088	.618	.284	3.381	**.001**	.866	3.310
		R = .284 R^2^ = .081 F = 11.430 p = .000*							
**Total PSS**	3	(Constant)	30.406	.681		44.67	**.000**	29.059	31.752
		Gender (Female)	2.063	1.015	.172	2.033	**.044**	.055	4.071
		Other chronic diseases (No)	3.429	1.133	.261	3.027	**.003**	1.187	5.671
		Smoking status (I never drank)	2.729	.908	.252	3.005	**.003**	.932	4.526
		R = .381 R^2^ = .145 F = 7.22 p = .000*							
**Total DAS**	4	(Constant)	2.751	.961		2.863	**.005**	.850	4.653
		Marital status (Married)	1.978	7.04	.225	2.810	**.006**	.585	3.372
		Employment status (No)	2.217	.583	.304	3.802	**.000**	1.063	3.370
		Duration of COPD (11 years or more)	–1.435	.607	–.188	2.365	**.020**	–2.635	–.234
		Getting education about the COPD (basic information about COPD) (No)	1.162	.573	.164	2.026	**.045**	.027	2.296
		Dyspnea symptom (No)	1.440	.620	.187	2.323	**.022**	.213	2.667
		R = .478 R^2^ = .228 F = 7.456 p = .000*							

* Significance level was accepted as p < 0.05; Abbreviations: CI, confidence interval; SE, standard error; β, standardized regression coefficient; PSS: Perceived stress scale; DAS: Death anxiety scale.

When the patients’ PSS and DAS mean scores were analyzed, it was found that perceived stress positively and statistically significantly predicted death anxiety (F = 4.332, p < 0.05) (Table 4).

**Table 4:** Regression analysis results regarding death anxiety being explained by perceived stress – Siirt, Southeast Region, Turkey, 2022.

Independent variables	Unstandardized coefficients		Standardized coefficients	t	Sig.	95,0% CI	
	B	SE	*β*			Lower bound	Upper bound
**Total PSS**	.115	.055	.180	2.081	**.039**	.006	.224
R = .180 R^2^ = .032 F = 4.332 **p = .039**							

Abbreviations: CI, confidence interval; SE, standard error; β, standardized regression coefficient; PSS: Perceived stress scale.

## DISCUSSION

COPD is a challenging disease that threatens life, gets the individual to lose his/her autonomy, causes important activities and relations to change, and requires both physical and mental adaptation. In addition to physio-pathological symptoms such as dyspnea, cough, and fatigue experienced, patients are severely affected by many mental problems such as stress and death anxiety. It is important to meet the physical needs of COPD patients as well as provide them with mental support^(8,11)^.

The study conducted to determine perceived stress and death anxiety levels in COPD patients and to identify the relationship between them, found that the patients experienced high levels of stress (Table 2). In the study conducted by Yohannes et al.^(19)^, 25% of COPD patients were reported to be prone to stress. In another study, it was reported that the patients did not know how to change their lives, and that therefore, they were prone to experiencing stress^(8)^. In another study, it was found that there was a decrease in the stress level, as well as in the depression and anxiety symptoms experienced by COPD patients who applied virtual reality^(20)^. Stress has been shown to be associated with significantly higher rates of depressive symptoms and poorer quality of life in individuals with COPD than in those without COPD^(21)^. These findings are in parallel with those obtained in the present study. As COPD is a chronic disease, the necessity for the patients to cope with the problems they experience may have increased their stress levels.

In terms of sex, it was found that female patients were more affected (Table 3). In the study conducted by Nal et al.^(9)^, it was reported that sex affected death anxiety, and that females experienced higher levels of stress compared to males. In the study conducted by Aghaei et al.^(22)^, it was found that women mostly experienced perceived stress situations. In a study conducted on different disease groups, stress levels perceived by women were higher than those of men^(23)^. It can be stated that as the responsibility for housework is on the shoulders of women in the Turkish society^(24)^ and women are more emotional compared to men, women’s stress levels are higher.

It was determined that in terms of marital status, married patients were affected by perceived insufficient self-efficacy (Table 3). It can be claimed that due to high expectations regarding spouse roles according to socio-cultural structure and the obligation to carry out both domestic and social roles^(24)^, married individuals had higher levels of perceived insufficient self-efficacy.

It was found that as the duration of the disease increased, the patient’s perceived insufficient self-efficacy levels and perceived stress/distress levels increased (Table 3). In the literature review, no relationship was found between the duration of the disease and perceived insufficient self-efficacy. It can be stated that the finding in the present study may have resulted from the uncertainty about the disease process and occurrence of symptoms as the disease progressed.

It was determined that those who had other chronic diseases had higher levels of stress (Table 3). In the study conducted by Xu et al.^(23)^, perceived stress levels of individuals with chronic diseases were found to be high. In a study conducted in the COVID-19 pandemic period, perceived stress levels were found to be high in patients with chronic diseases^(20)^. A similar result was obtained in the study conducted by Bayrak et al.^(7)^, and a positive and significant relationship was found between having a comorbid disease and perceived stress levels. It can be inferred that patients’ stress levels increased due to occurrence of symptoms of the other disease in addition to the existing disease and because they were insufficient in terms of coping with problems related to these.

It was found that the patients who did not smoke were under stress (Table 3). In a study conducted, an inverse correlation was determined between active smoking and anxiety symptoms^(8)^. Situations that lead to stress are discomforting, and the patients try to eliminate this discomfort. Smoking is described by the patients as a behavior to reduce stress^(25)^. In our study, the perceived stress of non-smoking patients was found to be significant, indicating that they were unable to cope with stress compared to smokers.

The patients were found to experience a moderate level of death anxiety (Table 2). In a similar study conducted by Bülbüloğlu and Kaplan Serin^(4)^, it was determined that the patients experienced a high level of death anxiety. In a study conducted on older individuals with COPD, it was reported that most of the patients lived death anxiety^(9)^. In other studies conducted, moderate levels of death anxiety were found among the COPD patients^(11,26)^. In another study conducted, it was stated that the patients knew that the symptoms they experienced were related to their disease, and that as the symptoms increased, their death anxiety increased^(11)^. It has been observed that patients have frequent complaints of distressed and difficult breathing and that they perceive this symptom as a serious threat to their lives^(11)^. In our study, the fact that the majority experienced dyspnea symptoms may have led to an increase in death anxiety. The reason why this situation spreads death anxiety can be explained by the fact that respiration is the most fundamental vital sign.

It was determined that in terms of marital status, most married patients experienced death anxiety (Table 3). As high responsibilities of married individuals in the Turkish society in home-child care^(24)^ can cause them to feel responsible for those left behind after their death, this may have led them to experience death anxiety.

Unemployed patients were found to have been more affected by death anxiety (Table 3). In the study conducted by Şahan et al.^(27)^ on patients who had myocardial infarction, death anxiety scores of unemployed patients were found to be significantly higher than those of employed patients. Working life and having an occupation keep employed individuals’ minds busy and make them more active and social, thus making them accept death more easily, which results in reduced death anxiety.

It was found that as the duration of the disease increased, so did death anxiety in the patients (Table 3). In a study similar to the present one, COPD patients were determined to have experienced higher levels of death anxiety as the duration of the disease increased^(9)^. Patients’ failure to manage symptoms as the duration of the disease increased may have triggered death anxiety related to the disease.

The study showed that the patients who did not receive training on the disease had their death anxiety increased (Table 3), which may be a result of their limited awareness of the disease. In the literature review, no study analyzing the influence of training on COPD and death anxiety.

It was determined that the patients who experienced dyspnea syndrome had high levels of death anxiety (Table 3). In a similar study conducted by Bülbüloğlu and Kaplan Serin^(4)^, death anxiety was reported to be high in COPD patients with high perceived dyspnea. In another study conducted, it was stated that shortness of breath experienced during the exacerbation of COPD caused patients to feel a near-death experience^(28)^. In yet another study, it was reported that the patients perceived distressed and labored breathing as a serious source of threat for their lives^(11)^. Dyspnea is a primary problem in COPD. Many symptoms experienced lead to restrictions in patients’ daily living activities. These symptoms have been reported to lead to an increase in death anxiety levels in the patients^(4,12)^. In other studies conducted, the majority of the patients were found to experience shortness of breath^(1,29)^. Hence, it can be claimed that the multitude of symptoms experienced cause the patients to see these symptoms as a serious source of threat to their lives. In line with these findings, it can also be stated that the symptoms seen in the patients are a cause of increase in their death anxiety.

It was observed that the patients’ perceived stress levels affected their level of death anxiety (Table 4). The attacks they experience, shortness of breath, phobias such as not being able to breathe again, significant changes in their lives regarding future, and ideals and targets in their lives increase death anxiety among COPD patients^(26)^. In a study conducted in the COVID-19 pandemic period, factors related with daily routine changes were reported to be a significant stress factor in the patients. It has been stated that these stress factors could increase the exacerbation of the disease and death risk in individuals^(5)^. The complicated symptoms seen in COPD patients and their inability to cope with stress can increase the death anxiety experienced.

## Limitations Of The Study

The study has some limitations. The perceived stress levels of patients are limited by the Perceived Stress Scale developed by Eskin et al.^(16)^, and the levels of death anxiety obtained using the Death Anxiety Scale developed by Akca and Kose^(18)^. Another limitation of the study is that it was conducted in a single hospital in the province, with the participation of COPD patients who agreed to participate. Therefore, they cannot be generalized to all patients and the reliability of the data is limited to the accuracy of the answers given by the patients who participated in the study.

## CONCLUSION

In conclusion, it was determined in the study that COPD patients had a high level of perceived stress and a moderate level of death anxiety. It was also found that perceived stress level affected death anxiety level. It can be claimed that COPD patients are prone to stress that could directly affect their general health, and that this situation leads to the development of death anxiety. In this context, increase in the awareness of COPD patients about the disease and the development of national policies for the prevention of the disease and effective intervention can be recommended. In addition, an evaluation and support of COPD patients in the treatment and care process, not only physically but also mentally and spiritually, should also be considered.
